# Lessons from the Criticality of the Spanish High Capacity Road Network on Direct, Representative Democracies and Technocracies

**DOI:** 10.1007/s12061-022-09451-5

**Published:** 2022-06-20

**Authors:** Juan Carlos Martín, Concepción Román

**Affiliations:** Department of Applied Economics, Institute of Tourism and Sustainable Economic Development, Universidad de Las Palmas de Gran Canaria, 35017 Las Palmas de Gran Canaria, Spain

**Keywords:** Federalism, Roads’ criticality, Complex networks, Accessibility DEA indicators, Technocracy, Direct and representative democracy

## Abstract

This paper aims to shed some light on an issue that has not been analyzed enough in previous studies on complex transportation networks. The financial crisis and disrupting events like the COVID-19 pandemic episode are affecting how governments make crucial decisions regarding policymaking paying more attention to experts’ opinions. The impacts of disruptive events that could affect each road section will be analyzed using the criticality of the high capacity road network in Spain under different policymaking scenarios, such as direct democracy based on pure provincial decentralization (federal or cantonal vision), representative democracy in which decisions are taken by the National Parliament, and the governance based on technocracy stimulated by ‘what matters is what works’ (Southern *Local Economy*, *16*(4), 264-271, 2001) using an Evidence-Based Policy Making (EBPM) case study based on a Data Envelopment Analysis applied to four accessibility indicators. We will complement our analysis with the different results obtained by the different national parties that were represented in the past election (November, 2019): PSOE, PP, VOX, Unidas Podemos, Ezquerra Republicana de Catalunya, Ciudadanos, Junts per Catalunya, Partido Nacionalista Vasco and Euskal Herria Bildu, exploring in-depth the obtained differences between the technocrat solution and the direct and representative democracy results. Important insights and lessons for the future will be obtained from the different party visions observed among the regionalist (nationalist) and the state-wide parties. The spillover effects created by the networks are so important that the room for federalist solutions might be very limited.

**Supplementary Information:**

The online version contains supplementary material available at 10.1007/s12061-022-09451-5.

## Introduction

Spain is usually advocated as an example of a peaceful transition from dictatorships to democracies after the death of Franco (the dictator that ruled Spain for almost 40 years after the end of a bloody civil war in 1936–39) (Hopkin, 1999). The national parliament of the young democracy (350 congressmen) was mostly concentrated in the hands of two political parties that have been forming governments since free elections were possible in the country: (1) a center-left party (Partido Socialista Obrero Español –PSOE); and (2) a center-right party (first Unión de Centro Democrático –UCD-, then Alianza Popular –AP- and now Partido Popular -PP) (Gunther et al., 2004; Hopkin, 2005). In the 2008 elections, 323 congressmen were in hands of these two main national parties (92%). The rest of the parliament was in hands of two small national parties (Izquierda Unida –IU- and Unión Progreso y Democracia -UPyD) and several nationalist parties such as Convergència i Unió (CIU), Esquerra Republicana de Catalunya (ERC) or Eusko Alderdi Jeltzalea-Partido Nacionalista Vasco (EAJ-PNV).

The economic downturn and the perception of corruption provoked a collapse in the bipartisanship in the elections of 2015 (Bosch and Durán, 2019). Since then, three more elections, held in 2016, 2019-April and 2019-November, showed that the political party arch of the Spanish National Parliament was no longer dominated by the former once stable bipartisanship. This political period was hallmarked by: (1) the most important constitutional crisis marked by the Catalan independence movement since the attempted military coup on 23 February 1981; (2) the Spanish exceptionalism in the European Union (EU) was finally ended by the electoral support for a far-right party (Vox) (Turnbull-Dugarte, 2019); (3) the first coalition government in the young democracy (PSOE and Unidas Podemos) saw the light after the last election held in November 2019; and (4) the trend of a fragmented and polarized Congress started in 2015 continued in the last election and the government coalition does not have a majority in the chamber. Rodon (2020) presents the background of the turbulent period and analyzed the likely impacts in the years to come, contending that the years of bipartisanship and the lack of a successful far-right party in the parliament are over.

The EU is characterized because a number of policies are decided and implemented at different government layers (Vickerman, 2008). The multi-level governance between local, regional, inter-regional, national and supra-national governments makes that some policy decisions or evaluations usually ignore all the potential benefits that can be obtained by network externalities and spillover effects (Condeço-Melhorado et al., 2011; Gutiérrez et al., 2010). The territorial tension has also played a role at the time of passing national infrastructure investment plans (Vidal and Sánchez-Vítores, 2019) in which the support of ethno-regionalist parties was exchanged by bargaining with more regional autonomy or better investment deals in comparison with other Spanish regions. In summary, network externalities usually hinder the economic projects appraisals as it is problematic to calculate or allocate benefits and costs of the different spatial multi-level governments.

The course of territorial tension, the bargaining power of ethno-regionalist parties, the corruption cases of the former bipartisanship system as well as the doubts that generate the technical capacity of politicians, in general, has exacerbated the strong dilemma that exists between representative democracy, direct democracy and technocracy. Bertsou and Pastorella (2017) contend that the citizens’ attitudes towards technocracy and the idea of governance by unelected experts are still unexplored. The authors find that the levels of trust in current representative political institutions determine citizens’ preferences on technocracy and authoritarian historical legacies explain partly the significant cross-national observed differences. Nevertheless, Webb (2013) shows that technocracy can be compatible with citizens’ demand for more direct participation on specific political issues.

Font et al. (2015) show that citizen preferences are not unidimensional and for some issues, citizens prefer either to participate directly or to be represented by politicians or by technocrats. The authors conjecture that experts’ governance might include citizens who value that: (1) technocrats have a central role in government; (2) decisions should be based on efficiency and expertise instead of political bargaining processes; (3) fellow citizens or elected representatives are not prepared to decide over complex issues that are beyond their expertise. Similarly, Bertsou and Caramani (2022) find that citizens’ attitudes towards technocracy, direct democracy and representative democracy usually overlap. The authors discuss that citizens’ frustration with politicians and the increasing demand for technocrats open the room for alternative governance systems.

There are multiple examples that belong to the sphere of complex issues such as the decisions on transport infrastructures related to accessibility, vulnerability, resilience and criticality, to cite only a limited number of them. Martin (2012) provided four reasons to study resilience: (1) the impact of natural and man-made disasters; (2) the analysis of how different ecosystems respond to shocks; (3) economic results can be affected by major disruptions; and (4) financial and economic crisis affect very differently on distinct governance layers. Reggiani et al. (2015) interpreted for the first time the relationship between resilience and vulnerability in transport networks clarifying the distinction between the ecological and engineering approaches. The authors found that resilience and vulnerability in transport networks are highly affected by connectivity and accessibility.

Our case study will be based on the criticality of the road sections of the Spanish high-capacity road network which is split into 352 different sections. Thus, it is assumed that there is a budget or contingent task force that need to be allocated in the network in order to maintain it in operation preventing undesirable disruption or shutdowns. Thus, the four aforementioned issues are included in our study linking accessibility with criticality as a way to analyze the resilience of the Spanish high capacity road network. A number of scenarios that resemble direct democracy, representative democracy and technocracy will be analyzed in an Evidence-Based Policy Making (EBPM) study. Thus, it will be possible to compare the results obtained from different democratic systems, selection rules, and political elites, and how these diverge from the optimal solution. EBPM has been highlighted as the driving force and desire for democratizing the policy process (Clarence, 2002; Fontaine, 2020; Marchionni & Reijula, 2019). In fact, the proposal was advocated as a way to provide instrumental rationality to the policymaking process.

Thus, the aim of this paper is to show how the solution for each of the scenarios changes according to whether the decision is made by the citizens, congressmen or technocrats. The different scenarios range from pure citizens’ participatory system (extreme cantonal or federal vision), or a more centralized vision in which decisions are taken by the National Parliament (representative democracy) to the experts’ governance based on technocracy stimulated by ‘what matters is what works’ (Southern, 2001).

In addition, we also analyze the results from the perspective of the mainstream political elites seeing how the scenarios proposed by political parties that finally succeeded in forming a parliamentary group in the past election (November, 2019) resemble more or less the optimal solution. In concrete, the scenarios proposed by PSOE, PP, VOX, Unidas Podemos, Ezquerra Republicana de Catalunya, Ciudadanos, Junts per Catalunya, Partido Nacionalista Vasco and Euskal Herria Bildu will be compared with the optimal solution. We explore in-depth the observed differences between the technocratic solution with direct and representative democracy results as well as the results obtained by each parliamentary group. In this respect, the technocratic solution acts as the optimal solution that diverges more from other analyzed scenarios when the centrifugal forces of nationalist parties become more apparent.

## Literature Review

### Direct Democracy, Representative Democracy and Technocracy

Policymaking in modern societies is usually biased by experts’ opinions and advice. Scientific input is usually needed in multiple fields and cabinet members are not expected to have enough expertise in all the legislative issues. There are multiple policy debates that are necessarily tamed by critical scientific input (Chakraborty et al., 2020). However, it is not straightforward to incorporate experts’ input into policymaking. Bertsou and Caramani (2020) contend that expert roles in politics are multivariate through a continuum of possibilities that range from the pure government of experts (technocracy) to mere ‘advisors’ who adopt a subordinate role in which final decisions are in hands of the cabinet members who are usually traditional career politicians. Societies usually face an important dilemma between how to balance each type of citizens’ participation from direct democracy via referenda, representative democracy via government decisions or technocracy (Dommett & Temple, 2020).

Lavezzolo et al. (2021) argue that representative democracy has brought tension between responsiveness and responsibility, in the authors’ words, between representing citizens’ preferences and governing effectively. These two issues are aligned with the representative democracy redefinition advocated by Scharpf (2006) and Mair (2013) that is based on two main pillars, namely, output legitimacy (responsibility) and popular sovereignty (responsiveness). Populism and technocracy are the two antagonist political movements that have grown because of the dual tension between responsiveness and responsibility. Populism and technocracy are seen as the natural responses to the lack of responsiveness and responsibility of representative democracies (Caramani, 2017; Leininger & Meijers, 2021). Bertsou and Pastorella (2017) find that there is a negative relationship between trust in political institutions and preferences for technocrat governments.

The XIV legislature in the young Spanish democracy presided by the prime minister Pedro Sánchez has become the second largest in history in terms of the number of vice presidencies and ministries, and taking into account all the secretaries, undersecretaries and general directorates of the Presidency and ministries, the increase in the number of senior officials in Spain reaches 36%, from 190 to 260 (López-González, 2020). The cabinet has also increased the number of ‘advisors’, a figure that was created in the Spanish legislation when describing the composition of the personnel that assist senior officials. However, the critics have been more severe by the ‘hand-picked appointment’ than by the number increase. Civil servants bring to the Supreme Court the appointment of 26 senior government officials. The upper bodies of the Administration, grouped in Fedeca, have objected to the appointment to the Supreme Court, and Jordi Solé, one representative of the group manifested that “we can accept that the law opens a door to fill, in certain circumstances, some of the senior officials’ positions with people outside the Administration with recognized solvency and professional competence, but what we cannot accept is that this exception might be used with open hands" (Europapress, 2020).

As seen, expert advice incorporation into government cabinets is not exempt from criticism because ‘advisors’ are often perceived to be biased, either because they can be captured by some industries or lobbyists or because they are seen as personal with no enough reputation whose merits are more based on closed relationships with politicians rather than real and authentic technical competence. Nevertheless, Dommett and Pearce (2019) find that, in Western societies, citizens’ preferences show a relative consensus on the support for the involvement of experts in policymaking. Lavezzolo et al. (2021) show that ‘ceteris paribus, voters prefer party candidates with high technical expertise to ones without technical expertise’ (p. 7), using Spain as a case study. This result is in line with other studies that analyze the preferences towards experts’ government involvement, stealth democracy attitudes or technocratic attitudes (Font et al., 2015; Lavezzolo & Ramiro, 2018; Bertsou & Caramani, 2020). Lavezzolo et al. (2021) also comment on the existence of a tradeoff between responsibility and responsiveness with the experts’ government involvement (Blondel 1991; Alexiadou and Gunaydin 2019). On one hand, societies prefer experts in cabinet because they are seen as more competent and responsible, so their policy decisions provide necessary structural changes that are considered effective in the long-term and are not subject to short-term partisan electoral bargaining. On the other hand, experts are also seen as elites who live far away from reality, in no need to listen to the citizens’ demands, unaccountable and without the necessity of getting citizens’ approval.

### The Spanish High-Capacity Road Network Criticality

Jenelius and Mattsson (2015) contend that in order to prevent or restore the optimal conditions of the road network against events of congestion or network disruptions, the governments need to allocate resources as well as envisage contingent plans. After this study, networks resilience gained academic attention and numerous studies analyze how, when and where, networks’ disruption can be adapted in order to alleviate and minimize the potential economic loss (Do & Jung, 2018; Gauthier et al., 2018; Kaviani et al., 2017; Qiang and Xu, 2020). The quantitative methods to analyze the consequences of severe or moderate disruptions of the road transport network are usually based on Hansen accessibility measures that take into account the aggregated losses due to longer travel distances, higher travel times or connectivity impediments that could affect the normal participation in activities related to employment, shopping, entertainment, health care, and emergency services.

A number of previous studies have been focused on identifying road network critical sections (Bagloee et al., 2017; García-Palomares et al. 2018; Gauthier et al., 2018; Jenelius, 2009; Ortega et al., 2020), with the help of different methods that are mainly based on the estimation of the disruption costs caused by the loss of the normal use of each of the road section considered in the analysis. Much less attention has been paid to finding the key drivers that explain the criticality of each section, but, in recent years, more attention has been given to this issue. In this sense, a new taxonomy of criticality on road networks can be proposed over the basis of the events that cause the road network disruption such as snow or ice (Sullivan et al., 2019), floods (Helderop & Grubesic, 2019), terrorism (Thöns & Stewart, 2019), earthquakes (Zhou et al., 2019) and climate change (Ortega et al., 2020). Nevertheless, an ample majority of studies analyse road network criticality without explicitly mentioning the disruption cause.

## Data and Methodology

This section provides an overview of the data and the main methods used in the study to build the policymaking scenarios that will form the Evidence-Based Policy Making (EBPM) study. First, DEA is applied to a set of four partial accessibility indicators at the national and provincial levels to obtain a synthetic indicator that determines which road section is more or less critical using the national or provincial perspective. (2) The technocrat solution is determined by the vector obtained in the first step at the national level; (3) The dataset of voters and congressmen for each province and political party in Spain during the last election held in Spain in November 2019 will be used to obtain the critical road sections under two different plurality rules; (4) voters will be used to build the scenario of direct democracy and congressmen will be used to build the representative democracy scenario; (5) 10 constituency scenarios based on the set of the 47 peninsular provinces and on the set of provinces for which different political parties could conform a representative party at the National Parliament are also analyzed; and (6) two dissimilarity measures are proposed to compare the eighty different scenarios proposed with the technocrat solution.

### Data and High Capacity Road Network

**Fig. 1:**
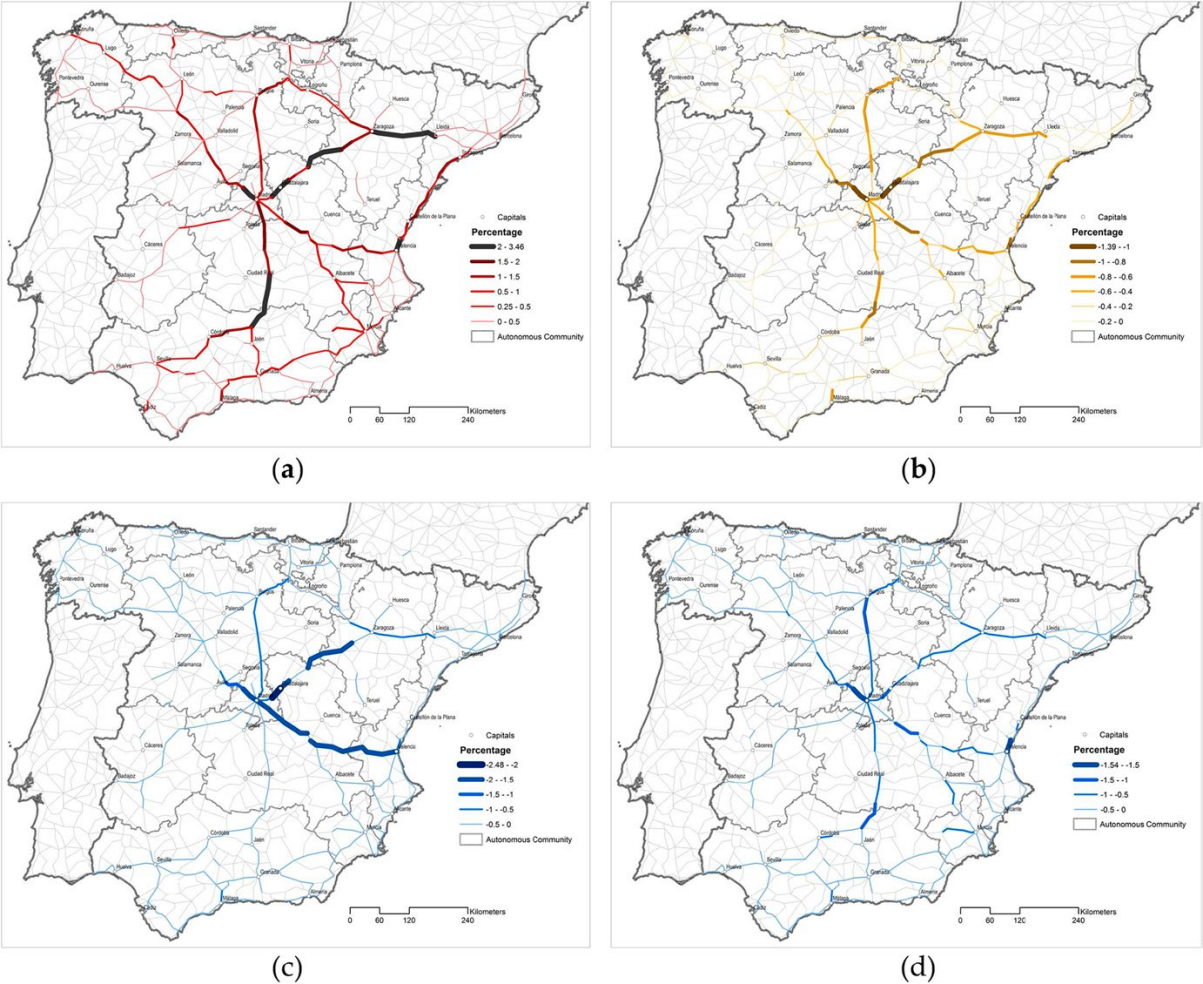
Critical sections of the Spanish high-capacity road: **a** Travel time increase; **b** Potential market loss; **c** Daily accessibility loss (3 h); **d** Daily accessibility loss (4 h)

The technocracy scenario is based on the results obtained by García-Palomares et al. (2018). A synthetic critical indicator is obtained by applying DEA to four partial accessibility indicators based on weighted travel time increases, weighted potential market losses and weighted daily accessibility losses within three and four hours’ thresholds (Cherchye et al., 2007). Figure 1 presents the partial picture of the criticality of each road section under each of the considered accessibility indicators, and it can be seen that each indicator highlights a different road section. A full description of the commonalities and the differences obtained by each accessibility indicator can be consulted in García-Palomares et al. (2018).

Before presenting how the scenarios for direct and representative democracy are going to be built, it is necessary to briefly overview the main features of the Spanish Electoral System that decrees how the Congress of Deputies is selected and conformed. The Congress has 350 seats proportionally distributed in 52 electoral constituency demarcations (50 provinces plus the autonomous African cities of Ceuta and Melilla). In the current study, the results will be based only on the seats of the provinces located in the Iberian Peninsula (325 seats), so the seats of the Balearic and Canary Islands plus those of the African autonomous cities are not included in the analysis. Thus, the imaginary parliament consists of 47 different provincial circumscriptions.

The analysis conducted in García-Palomares et al. (2018) is now repeated in the current study for each of the 47 provincial circumscriptions to obtain the critical road sections under each provincial vision. The main difference between the overall and the provincial analysis is that the accessibility indicators for each disrupted road section are calculated considering all the nodes and the potential interactions (47*46/2) or the provincial node that interacts with the rest of the nodes (46) respectively. Similarly, to the national case study, DEA is now applied to each of the provincial observations to calculate the critical road sections using only the provincial perspective. Thus, all the citizens in each province or the provincial congressmen will have the ranking of the critical road sections that determine uniquely the vote to select the critical road sections in the direct or representative democracy according to the values of the matrix *CI*_*ij*_.(i = 1 … 352, j = 1 … 47). The matrix *CI* determines the preference relation for each voter or congressman at the provincial level on the set of the alternatives (road sections). Thus a voter or congressman in the province *j* prefers road section *i* over road section *i’* if and only if *C*_*ij*_$\ge$* C*_*i’j*_*.* In this sense, the preference relation is determined on whether the road section is more or less critical to each of the provinces.

**Fig. 2:**
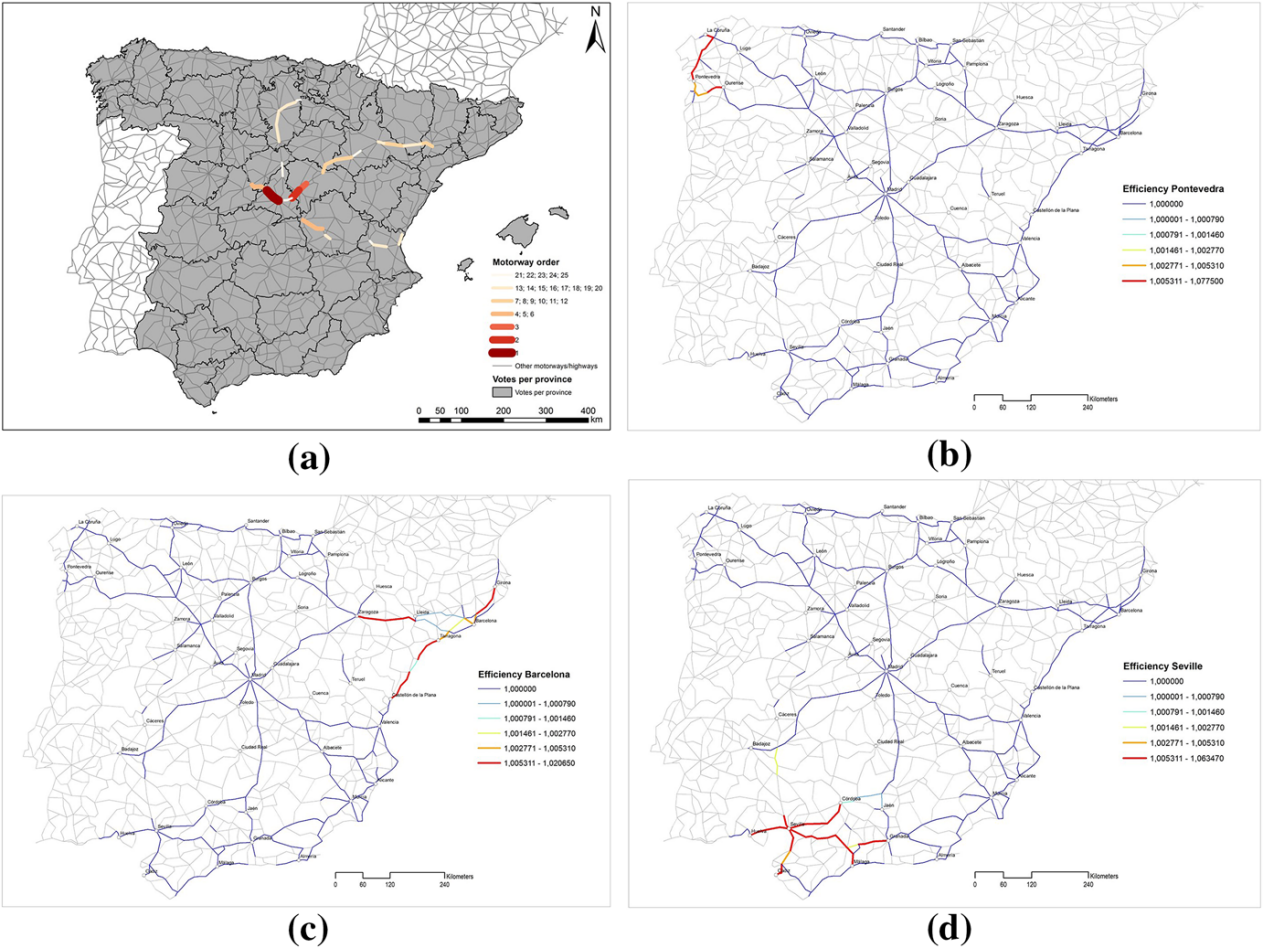
The 25 most critical sections of the Spanish high-capacity road: **a** Spain; **b** Pontevedra; **c** Barcelona; **d** Seville

Figure 2 shows the 25 most critical road sections in Spain (panel A) and the individual vision for three Spanish provinces namely Pontevedra (panel B), Barcelona (panel C) and Seville (panel D). The orange and red sections in each map are the most critical road sections for Spain and each of the provinces. Interestingly, it can be seen that some of the most critical road sections are not located within the boundaries of the province, and the number of critical road sections for each province is different. For example, Pontevedra has only 10 critical road sections in comparison with Barcelona and Seville which have 31 critical road sections respectively. The extreme cases are presented in Badajoz which has only one critical road section, and in Zaragoza, Valladolid and Madrid which have 55, 56 and 68 critical road sections respectively.

On the other hand, analysing now the most critical section for Spain which is located in the province of Madrid in the corridor Madrid-La Coruña, it is interesting to highlight that the section is only critical for 12 provinces in which the majority is represented by provinces of Castilla-León that are not very important in terms of voters and congressmen. Regarding the second and third most critical road sections, the two sections are critical for 13 and 7 provinces respectively. The two sections are located in the province of Guadalajara in the corridor Madrid-Barcelona, and without the support of Madrid and Zaragoza, the rest of the circumscriptions are relatively irrelevant. All this is important in a framework in which territorial imbalances are gaining a lot of media attention in Spain with the progressive acceptance of the concept of España vaciada (emptied Spain), and when the power of nationalist parties in the Parliament is now counterbalancing with the entry into the institutions of the electoral group Teruel Existe (Teruel also exists). Montes (2020) contends that the debate is not only limited to the Spanish case since it is well known that the European Union has traditionally prioritized territorial cohesion policy.

### Methodology

This section does neither cover the methods employed to calculate the individual accessibility indicators’ gain and loss nor the DEA methodology that calculates the synthetic criticality index for each road section. Interested readers are again referred to García-Palomares et al. (2018), but it is worth noting that the contra factual scenario is seen as the technocrat outcome and is based on the solution of the critical road network sections described in the previous study. The authors use a full network scan approach in order to calculate the criticality of the sections of the Spanish high-capacity network applying the data envelopment analysis (DEA) that synthesizes the information from four well-known accessibility indicators (average travel times, potential market and two daily contour accessibility indicators measured with three and four hours’ thresholds). Thus, the authors estimate a synthetic average accessibility loss due to each road section disruption that will be used as the optimal solution provided by technocrats that will be the reference point to compare other policymaking scenarios.

In this study, we focus on two important features that deserve a further discussion: the selection procedure to determine up to the 25 most critical road sections under direct and representative democracy regimes and the dissimilarity measure used to compare each of the solutions with the technocracy scenario (Fig. 2- Panel a).

#### Plurality Rule. 25 Rounds Plurality Rule. Different Constituencies Approach

The selection of the critical road sections under each scenario considered in the study is going to be approached as the selection of a society that consists of a finite number of individuals (voters for the direct democracy scenario and congressmen for the representative democracy scenario). In this case, each individual represented either by a voter or a congressman has a transitive preference over the set of alternatives (352 road Spanish sections) given by the road section criticality index discussed in Sect. 3. Thus, the plurality rule is a system in which each individual is allowed to vote for only one alternative as the most critical road section, and the section which gets more votes than any other counterpart is selected. In this study, we extend the plurality rule as follows: each individual votes for one road section and the set of the 25 road sections with the most votes is selected. We also analyse the plurality rule with 25 rounds (25 rounds plurality rule) in which there are 25 sequential voting plurality elections in which the set of the selected critical road sections in the *i-th* round is discarded from the set of alternatives in the *(i* + *1)th* voting. Advantages and disadvantages of plurality rules and other electoral systems such as majority rules are discussed in (Davis et al. 1972; Duverger, 1954; Niou, 2001).

As the Spanish Parliament is highly polarized and the government formation depends on nationalist parties of Cataluña and the Basque Country, the two selection rules are going to be further analyzed using ten different constituency scenarios formed by the 47 provinces of the Spanish Iberian Peninsula (Total) and the provinces of each of the 9 parliamentary groups. Thus the constituency scenarios are: (1) 47 Iberian peninsula provinces; (2) Partido Socialista Obrero Español (PSOE); (3) Partido Popular (PP); (4) VOX; (5) Unidas Podemos (UP); (6) Esquerra Republicana de Catalunya (ERC); (7) Ciudadanos (Cs); (8) Junts per Catalunya (JxCAT); (9) Partido Nacionalista Vasco (PNV); and (10) Eusko Halerria Bildu (EH Bildu). Thus, the results’ robustness will be assessed with the always compromising territorial issues that are present in the Spanish Parliament.

The territorial tension is always a relevant issue, and, for this reason, it is interesting to see to what extent the results obtained by the nationalist parties in Cataluña, ERC and JxCAT, as well as those obtained by the nationalist parties in the Basque Country, PNV and EH Bildu, are very different from other political parties that have more representation in the rest of the country. The subjacent idea of these scenarios is to preliminary analyze the effects of one of the three federal proposals that could change the current Spanish territorial model (Fossas-Espadaler, 2019): light federalism, federalism and asymmetric federalism. Thus, the main idea is to compare the optimal technocracy scenario with the one obtained by each of the nationalist parties in Cataluña or the Basque Country if their voters or congressmen could decide what road sections were the most critical. The results seem to point out the direction towards the lack of political and social consensus that will be aggravating the search for a satisfactory solution that could close definitively the territorial tension.

#### Dissimilarity Measures

In order to compare the different selections obtained in each of the 40 scenarios that are the result of having two different political systems, direct and representative democracy (voters and congressmen), two different rules to determine the critical road sections, and 10 different constituent provinces. All these issues were explained in the previous section, and now it is necessary to propose a dissimilarity measure that permits us to find which of the scenarios is closer to the technocrat solution. Each of the selections is characterized by being a structured set of 25 road sections from the total set of 352 road sections of the Spanish high capacity network as follows: $A=\left\{{a}_{1},\cdots ,{a}_{25}\left|{a}_{j}\in R\right.\right\}$ where $R=\left\{1,\cdots ,352\right\}$. The structure is given by the following two properties: (1) each element in A is different; and (2) *a*_*j*_ is more critical than *a*_*k*_ if and only if *j* is lower than *k*. For convenience, we denote the set of all the possible selections A in R as $\Phi (A,R)$, then we need to propose a dissimilarity measure on $\Phi (A,R)$ Interestingly, the 25 first elements of the ranking obtained at national level that conform the technocrat solution T belongs to $\Phi (A,R)$.

The first dissimilarity measure *d*_*1*_ is defined as a 25 components vector in which for each *i* we calculate the number of sections that do not appear in the first i-elements of T (the complementary to the number of sections that appear). In essence, this measure simply gives the number of segments that have not been selected and it resembles the overlapped coverage employed by Çakır et al. (2015) when the authors compare eighth global university rankings. For example, if we see that the sixth component of the measure is 4 then we know that four sections are not in the set of the six more critical road sections of the technocrat solution. More formally, for each *i* we define:

1 $${\mu }_{i}(j)=\begin{array}{c}1\text{\hspace{0.33em}}if\text{\hspace{0.33em}}{a}_{j}\in \left\{{t}_{1},\dots ,{t}_{i}\right\}\\ 0\text{\hspace{0.33em}}otherwise\end{array}\text{\hspace{0.33em}}\left|j=1\dots i\right.$$

Thus, we can now define d_1_ as:

2 $${d}_{1}(i)=i-\sum_{j=1}^{i}{\mu }_{i}(j)\text{\hspace{1em}}\left|i=1\dots 25\right.$$

This first measure is based on a criterion that does not take into account that a particular road section *a*_*i*_ does belong or not to a subset of T. This is a criterion of all or nothing that is the section should be or not selected by technocrats. However, as a complete ranking of the Spanish road network is known, *d*_*1*_ can be easily extended taking into account the degree of whether the selections are made with more or less error. The new measure is based on the Spearman’s Footrule distance (Yu et al., 2019). Now, we define the following auxiliary function:

3 $${\nu }_{i}(j)=abs(j-rank({a}_{i}))$$

where abs is the mathematical absolute value function and rank gives the position of each section according to the technocrats. Thus, we can now define d_2_ as:

4 $${d}_{2}(i)=\sum_{j=1}^{i}{\nu }_{i}(j)\text{\hspace{1em}}\left|i=1\dots 25\right.$$

## Results

Table 1 presents the results for the technocrat solution and the Total constituency scenario under direct and representative democracy, as well as one and 25 rounds selection rules. It can be seen that the most critical section according to technocrats (224) is seen very differently under each of the scenarios analyzed in the table. Thus, under direct democracy, the section is ranked as the seventh for the 1 round rule and the second for the 25 rounds rule. Meanwhile, under representative democracy, the section gets the fourth and the second position for one and 25 rounds rules, respectively.

**Table 1:** Road critical sections. Total constituency scenario

Technocracy	Direct Democracy				Representative Democracy			
	1 round		25 rounds		1 round		25 rounds	
Road Section	Road Section	Voters	Road Section	Voters	Road Section	Congressmen	Road Section	Congressmen
224	184	6832387	184	6832387	184	40	184	40
184	72	5609350	224	7915792	72	32	224	55
80	45	3317328	80	7532890	45	22	80	44
90	352	2547986	174	7587901	224	18	174	49
174	233	1939887	90	6578079	352	15	90	37
173	167	1641121	173	7587901	233	12	173	49
70	224	1337713	70	6666679	167	11	70	39
63	331	1238714	58	7532890	227	10	58	44
58	113	1149628	63	7532890	331	9	63	44
307	227	1137199	348	6578079	31	8	348	37
72	31	1119351	195	7088491	113	8	195	44
320	27	1028244	199	7088491	27	7	199	44
341	80	954811	342	7532890	80	7	342	44
352	275	941772	218	6832387	236	7	218	40
348	236	912075	352	9126065	275	7	352	52
321	68	795902	180	6935149	13	6	180	41
65	13	785240	350	6578079	68	6	350	37
195	314	761947	37	6578079	97	6	37	37
199	97	720592	38	6578079	208	6	38	37
193	239	709340	172	7097930	239	6	172	42
171	208	687391	197	6935149	252	6	197	41
342	252	676376	183	6920987	314	6	183	42
218	98	647554	206	6775301	65	5	206	40
180	120	580229	207	6775301	98	5	207	40
350	65	576898	216	6775301	120	5	216	40

Table 1 is constructed taking into account all the votes emitted by province in the last election held in November 2019 that will represent the direct democracy solution and the congressmen who obtained provincial representation that are used to obtain the representative democracy solution. It is assumed that voters and congressmen vote rationally according to the provincial solution provided by the DEA method applied at the provincial level.

Thus, attending exclusively to this most critical road section, we can conclude that representative and direct democracy under the 25 rounds plurality rule perform equally and better than the rest of the scenarios based on one round plurality rule. The worst solution is achieved by the direct democracy under one round plurality rule. It is worth noting then that if the society is interested in finding the most critical road section, the direct democracy under one round plurality rule will be the worst policymaking rule. Analyzing now the fourth and eighth columns of the table, we see that the selections provided by the direct and representative democracy under the 25 rounds rule are the same. On the other hand, it is also interesting to remark that the selection of the most critical road section under direct and representative democracy under the two plurality rules analyzed is the second most critical road Sect. (184).

A similar analysis can be done for the second and the third most critical sections. For example, the second most critical road Sect. (184) is seen equally for the four scenarios included in the table. In this case, there is not any difference between the decision rule or the democratic system that is employed to obtain the solution. The third most critical Sect. (80) presents a more heterogeneous picture. In this case, there are only two different rankings: 13^th^ for the one round rule and 3^rd^ for the 25 rounds rule. The democratic system employed does not have any effect for this section. Unfortunately, the picture is not so stable for other sections. For example, the fourth most critical Sect. (90) will not be selected by the one round rule. For the road Sect. 307 (the 10^th^ most critical section), the results are even worse than the previous commented ones, as this section will not be selected under any policy scenario.

Analyzing now the percentage of citizens who votes for each road section under each scenario, it can be seen first that plurality rules exhibit a very different pattern. For example, only the first two critical roads achieve a percentage higher than 10 when the one round rule is performed. Meanwhile, if the 25 rounds rule is applied then the percentages increase in all the cases, and the level of votes is always higher than 15 per cent. The patterns for the results of the representative democracy are very similar, but now the figures decrease as in the one round rule there is only one road section (the most critical one) which presents a percentage of the congressmen higher than 10 per cent. Meanwhile, the 25 rounds rule shows that in all the cases, the percentages of the congressmen in favour of each road section is always higher than 11 per cent.

It is evident that the analysis cannot be based on such one-to-one critical road sections comparison for each constituency scenario. For that reason, the dissimilarity measures will be used as a way to extract to what extent the different scenarios are more or less similar to the technocrat solution. Table 2 shows the dissimilarity measures for the 25 most critical segments according to different selection rules and democratic systems for the case of the total constituency scenario, remarking that a similar table can be constructed for each of the other constituency scenarios.

**Table 2:** Dissimilarity measures. Total constituency scenario

Technocracy	Direct Democracy				Representative Democracy			
	1 round		25 rounds		1 round		25 rounds	
Road Section	DM1	DM2	DM1	DM2	DM1	DM2	DM1	DM2
224	1	2	1	2	1	2	1	2
184	1	43	0	3	1	43	0	3
80	2	100	1	37	2	100	1	37
90	3	211	1	40	2	101	1	40
174	4	312	2	103	3	212	2	103
173	5	340	2	107	4	313	2	107
70	5	341	3	184	5	341	3	184
63	6	367	4	329	6	376	4	329
58	7	413	5	473	7	402	5	473
307	8	448	6	567	8	432	6	567
72	9	478	7	609	9	478	7	609
320	10	515	8	652	10	515	8	652
341	11	549	9	817	11	549	9	817
352	12	702	9	830	12	614	9	830
348	13	767	10	941	13	767	10	941
321	14	870	11	991	14	886	11	991
65	15	989	12	1119	15	989	12	1119
195	15	996	13	1253	16	1063	13	1253
199	16	1070	14	1386	16	1073	14	1386
193	17	1185	15	1413	17	1188	15	1413
171	17	1195	16	1474	18	1330	16	1474
342	18	1337	17	1541	18	1337	17	1541
218	19	1391	18	1625	18	1342	18	1625
180	20	1420	19	1708	19	1396	19	1708
350	20	1425	20	1797	20	1425	20	1797

DM1: Dissimilarity measure 1. DM2: Dissimilarity measure 2.

**Fig. 3:**
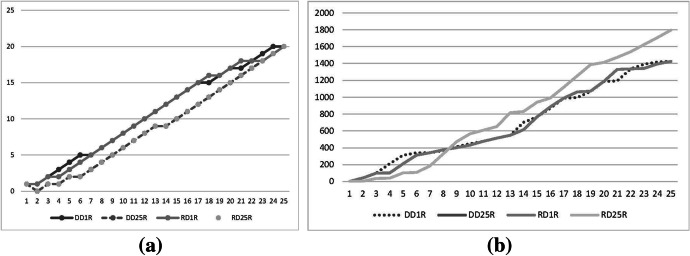
Critical Roads comparison. Total constituency scenario. **a** Measure 1. **b** Measure 2

The Spearman rank correlation of Table 2 shows that there are four different rankings with respect to the dissimilarity measures: 1 round rule-direct democracy-first dissimilarity measure, 1 round rule-representative democracy-first dissimilarity measure, 25 rounds rule-first dissimilarity measure, and second dissimilarity measure. In fact, it can be seen that the first dissimilarity measure presents the same results if the one round rule is applied independently of the democratic system used. The same applies to the second dissimilarity measure when the 25 rounds rule is applied. For this particular constituency scenario, it seems that the results based on the second dissimilarity measure are more stable. Figure 3 represents the dissimilarity values for the eight scenarios presented in Table 2 split in two panels: (a) first dissimilarity measure; and (b) second dissimilarity measure.

Figure 3 shows that both dissimilarity measures are highly correlated as they both measure the distance between the selection obtained in each scenario and the counterfactual solution provided by the technocrats. It can also be observed that there are only three different graphs for each of the measures as when the 25 rounds rule is applied the dissimilarity measures are equal for direct and representative democracies. Nevertheless, analyzing each individual measure, we see that the 25 rounds rule dominates the graphs in panel (a) as, independently of the number of critical roads chosen, the graph is always the lowest of all the shown graphs. The same result cannot be concluded in the case of the second dissimilarity measure shown in panel (b), as in some cases the lowest graph is represented by one of the three different graphs. Thus, it can be seen that up to the eighth most critical road section, the society will be better off by the 25 rounds rule; when the number of road sections goes from 8 to 15, the society is better off with one round rule and representative democracy; from 16 to 21 the society is better off with one round rule and direct democracy; and finally, from 22 to 25, the society is again better off with one round rule and representative democracy. Nine additional figures were obtained and can be consulted as supplementary material for each additional constituency scenario. There are interesting results that can be highlighted: (1) the national parties show a similar trend commented previously in the case of the total constituency scenario in which for the top critical road sections it seems better to leave the solution to the 25 rounds rule; (2) Unidas Podemos is the only political party for which the four graphs for each dissimilarity measure is observed, so in this case, the representative and direct democracy diverge in the selection of the critical road; (3) the second dissimilarity measure for the nationalist parties changes the values abruptly in comparison with the rest of the cases; and (4) the 25 rounds rule produces the better results for the society when the solution is selected according to the nationalist parties preferences.

**Fig. 4:**
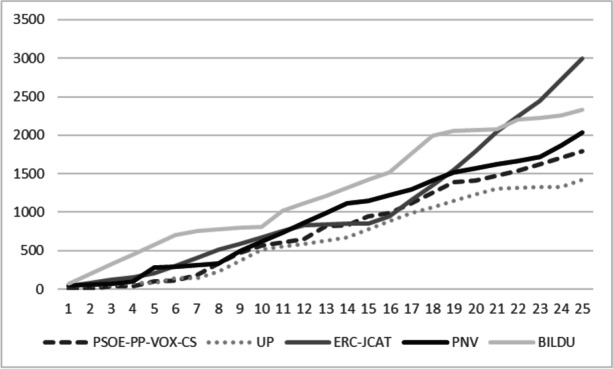
Second dissimilarity measure. 25 rounds rule and representative democracy. Political parties’ constituency scenarios

Figure 4 shows the results of the dissimilarity measure 2 when the 25 rounds plurality rule and representative democracy is applied for the selection of the critical road in the case of the political party constituency scenarios. First, it is interesting to remark that four state-wide parties like PSOE, PP, VOX and CS present the same exact results. It can be seen that at the left side of the figure, the national parties PSOE, PP, VOX and Cs produce the best results for society. Nevertheless, above seven critical road sections, the best solution is achieved if the solution were in hands of Unidas Podemos. In any case, it can be seen that the solutions are really bad in comparison with the technocrat solution.

## Discussion and Conclusions

Clarence (2002) contends that the search for good governance or co-governance can be delusional, and that fashion trends play a key part. The messiness of the process has been a dynamic constant that politicians have minimized with the help of experts or technocrats who determined ‘what needs to be done’. The current paper discusses how the optimal policies on the selection of the 25 most critical road sections are affected by the governance type of the triad that is represented by direct democracy, representative democracy and technocracy.

The current study stands in the strand of the EBPM literature that sheds light and evidence on how perfect rational policymaking at provincial layer can distort the optimal solution represented by technocracy. The divergences between technocracy and other policy scenarios that resemble either more direct citizen participation (direct democracy) or representative democracy have been studied with 80 different cases represented by: (1) two pluralist selection rules (one round and 25 rounds); (2) 10 constituency commissions; (3) two democratic systems (direct and representative democracies); and (4) two dissimilarity measures.

In this sense, the study is relevant to policymakers providing some insights that can be used by the main stakeholders of the National Spanish Parliament, namely the ethno-regionalist-nationalist parties’ vision (ERC, PNV, JxCAT and EH-BILDU), the national parties that form part of the governmental coalition (PSOE and UP) which want to move on what is known as an asymmetric federal country, the national parties which still display significant faith in the Autonomous Communities (PP and Cs) and VOX than wants to curtail some of the transfers given in the past to the Autonomous Communities centralizing again some of the public services, mainly health, education and state security forces. Our results are conclusive regarding that policymaking should give more vigour to ‘technocracy’ showing more faith in research and the contribution it can make to make the society better-off.

Our results show that independently of the dissimilarity measure, there is an important divergence obtained between the technocrat solution and the rest of the scenarios. It is particularly relevant that the divergence is more acute when the number of critical road sections is increased. It is not a surprise that the divergence is more important when the constituency commission is given to the nationalist parties. The results are grounded on the fact that voters and congressmen at provincial level are incapable of internalizing the important spillover effects that exist in road networks (Gutiérrez et al., 2010; Laird et al., 2005; Stepniak & Rosik, 2013). Similarly to what Bradford (2020) finds for the EU, it can be concluded here that the narrative for internalizing the network externalities should not be undermined. In the context of vulnerability and resilience, it can be concluded that any other solution that the technocrat will cause important accessibility losses if the resources are allocated by other private interests rather than by the evidence shown by well-adjusted transport models.

The EBPM agenda is certainly based on the science and research that shed evidence at the time of policies design that can work. Our analysis shows that it is possible to understand how different selection rules, constituency commissions, democratic systems and dissimilarity measures have an impact on each of the scenarios’ solutions. We analyzed how the solutions in each of the scenarios changed, and what was optimal for one scenario may not be optimal in another. In addition, we found that some solutions were the same. Moreover, we found that above four critical road sections, all the scenarios highly diverged from the technocrat solution. For that reason, in the interest of using rational policymaking, it is necessary to depoliticize the policy process. Clarence (2002) concludes that policy decisions should never be based on political beliefs or ideology but instead upon rational evidence and research. In the study, important insights and lessons for the future have been obtained from the different party visions observed among the regionalist (nationalist) and the state-wide parties. We find that the spillover effects created by the road network are so important that the room for federalist solutions seems to be very narrow. In this respect, it seems that the long-standing debate between centralization and decentralization in transport networks can be closed in favour of the central technocrat solution.

Some suggestions are left here as how this study could open new future research lines: 1) to analyse whether the Autonomous Communities as intermediate layers between the provinces and the country could moderate the bad results obtained; 2) to propose new dissimilarity measures that could normalize the results that limit the effects of the number of critical road sections that can be selected; and 3) to perform an econometric model that could provide more insights about the effects of each of the variables included in the scenarios.

## Supplementary Information

Below is the link to the electronic supplementary material.

Supplementary file1 (PDF 34 kb) Figures similar to Figure 3 are available online for each of the constituency scenario. See the Appendix.
