# Supplementary figures and images for: Lessons from the Criticality of the Spanish High Capacity Road Network on Direct, Representative Democracies and Technocracies
Source: Appl Spat Anal Policy. 2022 Jun 20:1–21. Online ahead of print. doi: 10.1007/s12061-022-09451-5 (PMC9206893; doi:10.1007/s12061-022-09451-5)

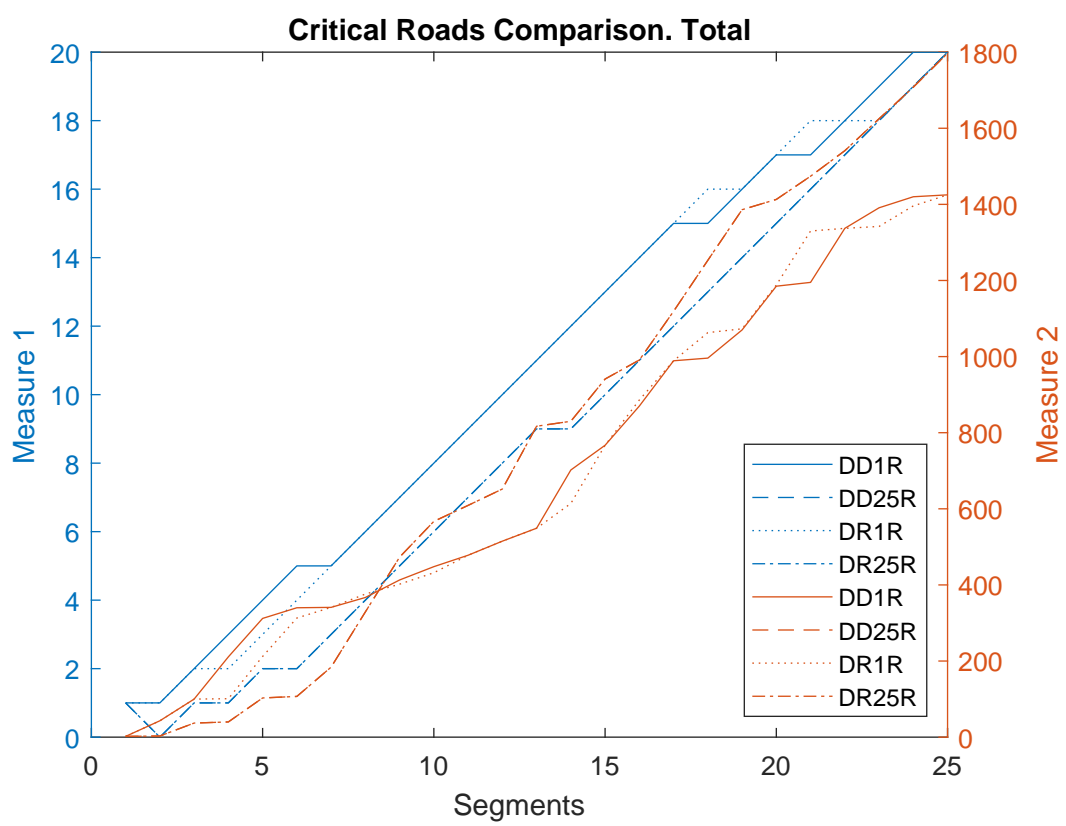

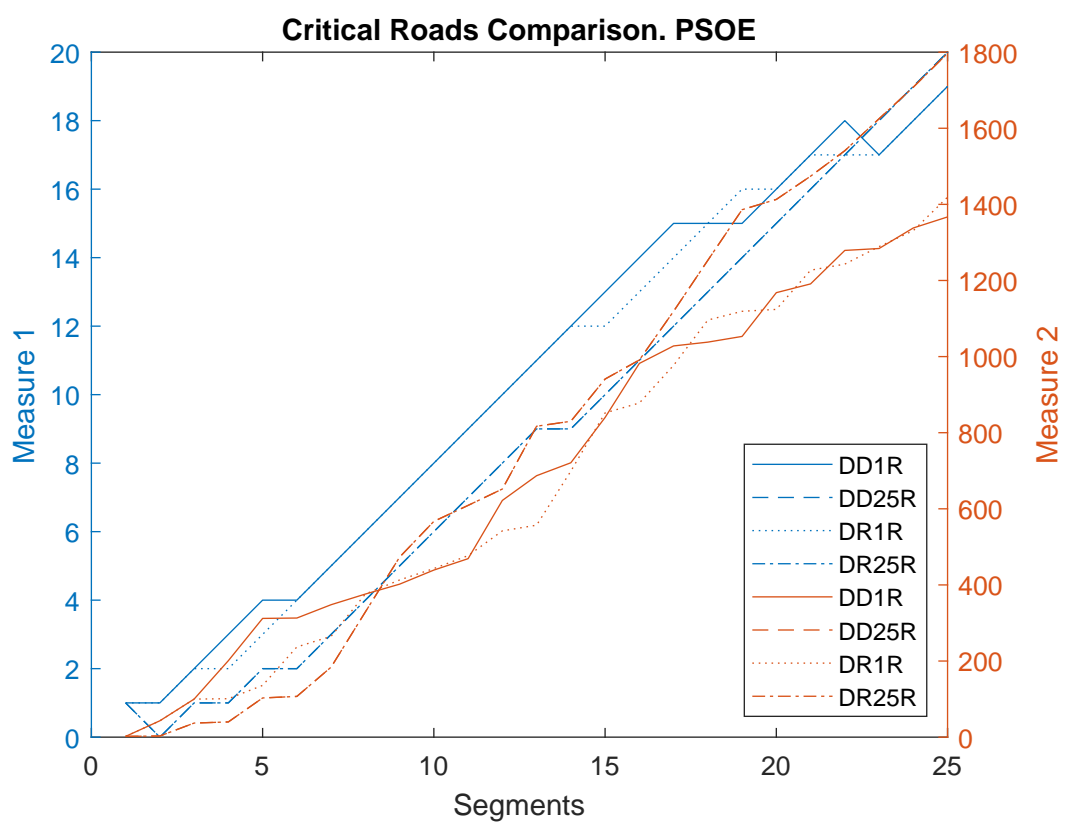

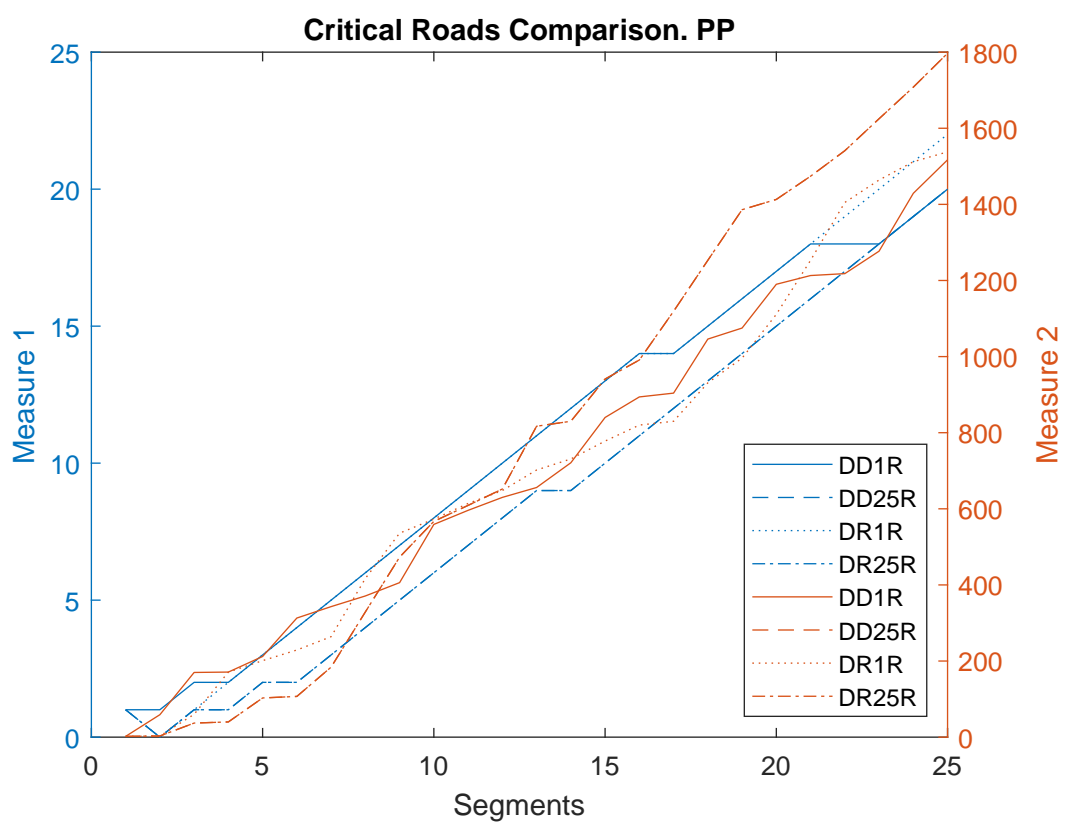

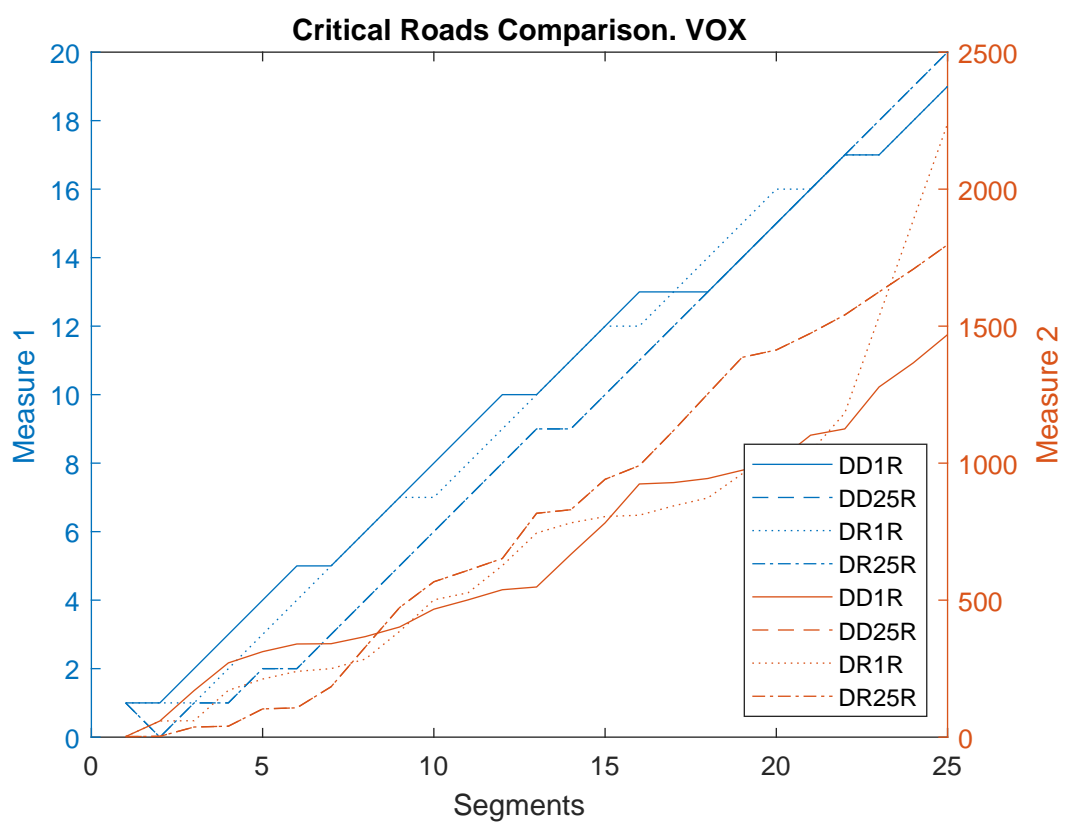

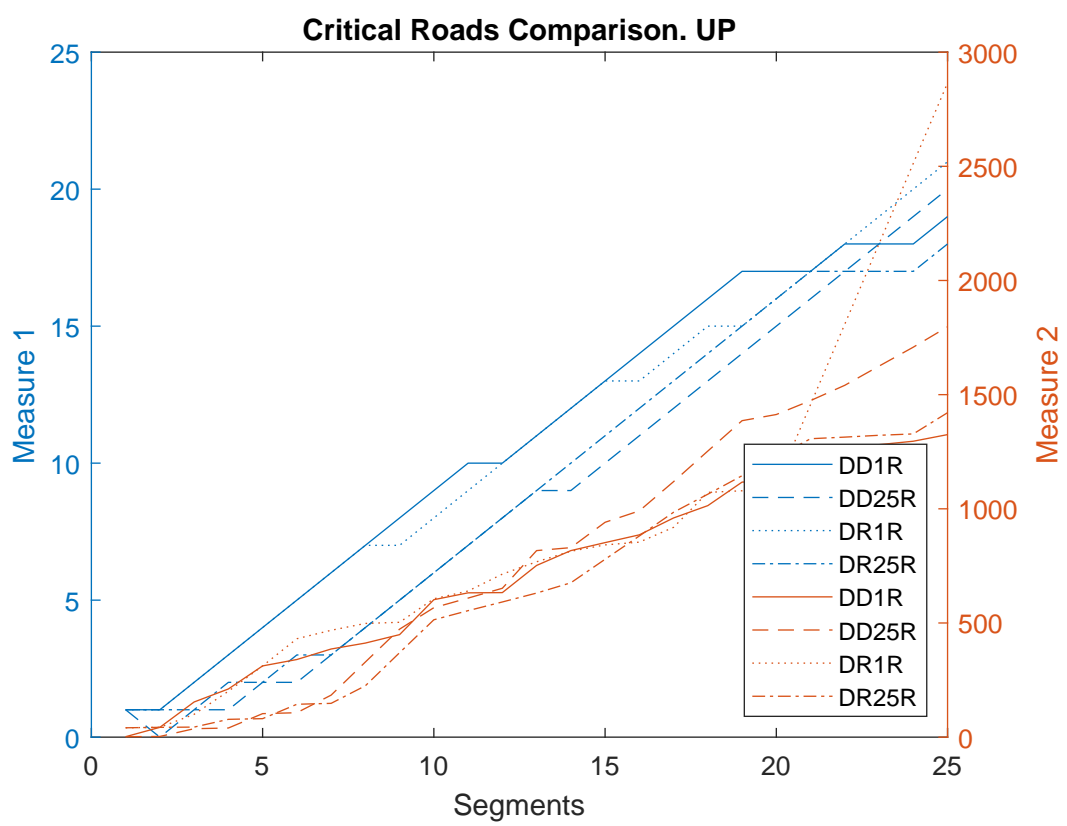

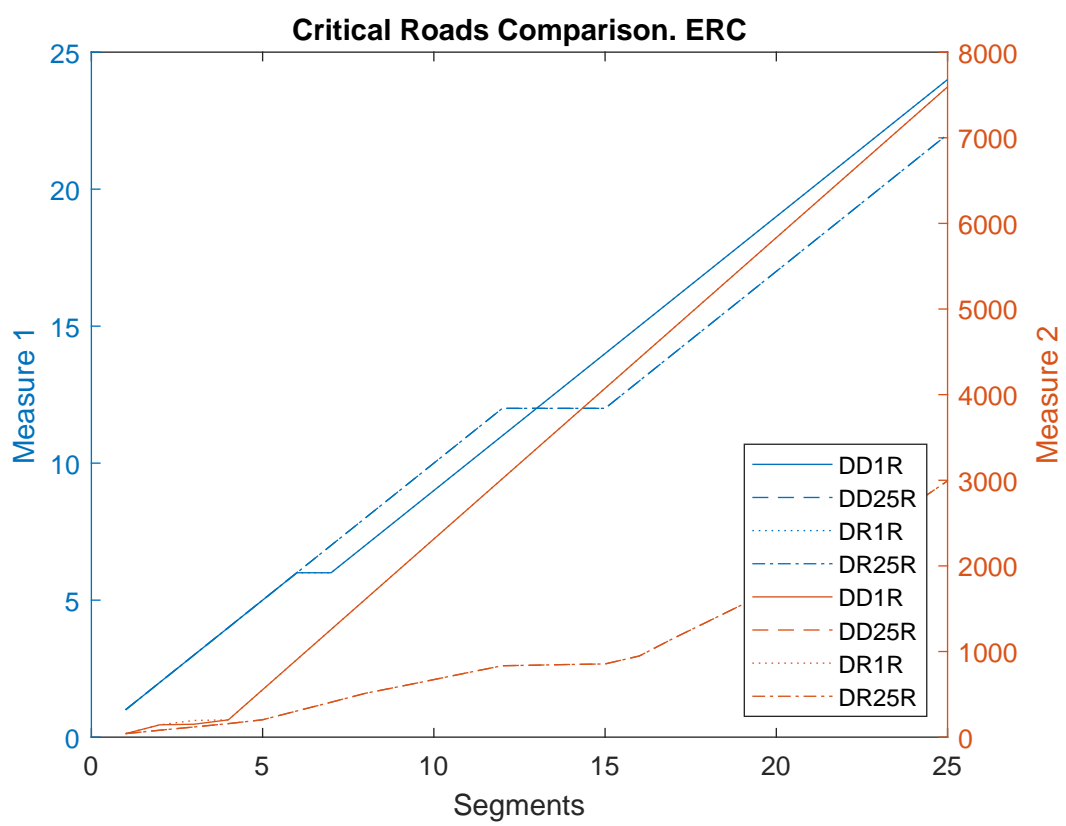

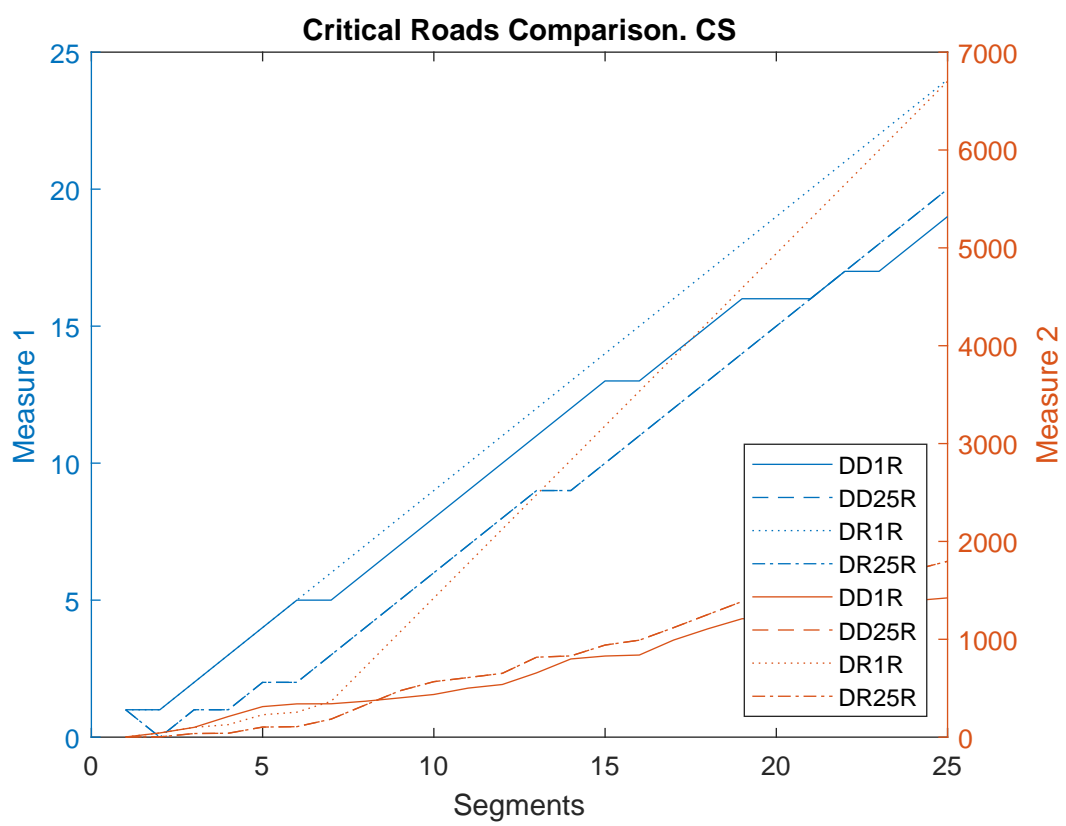

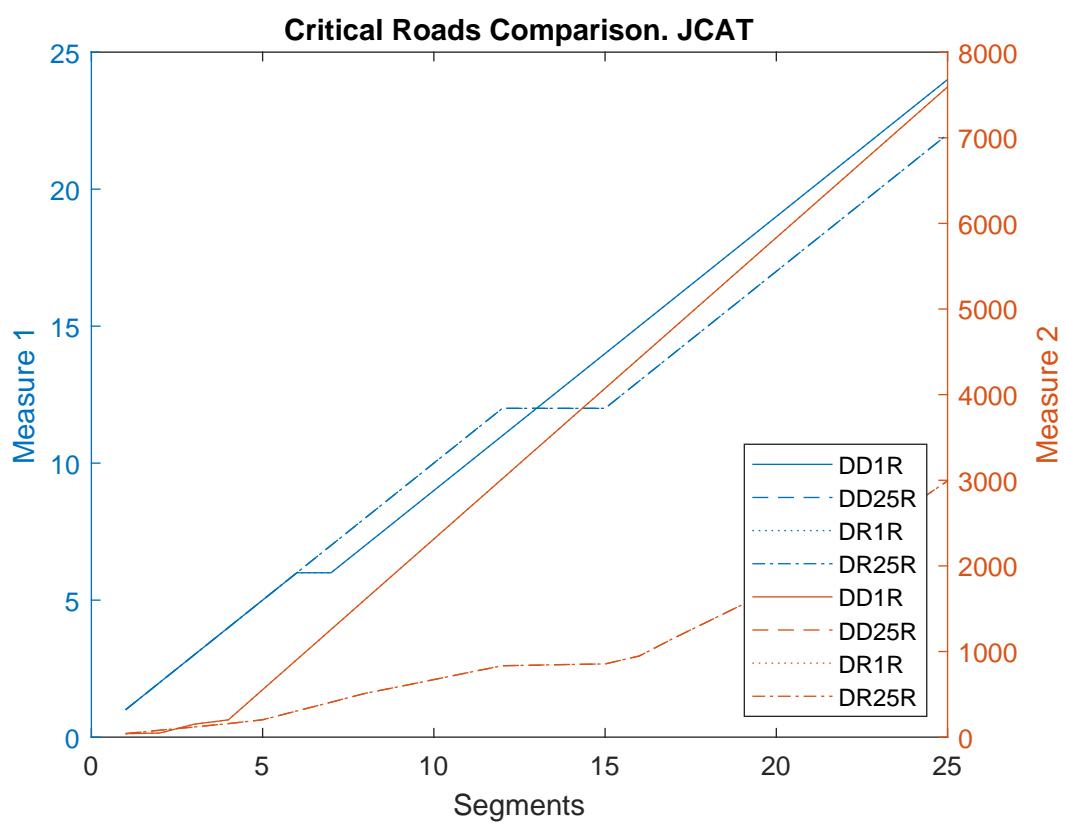

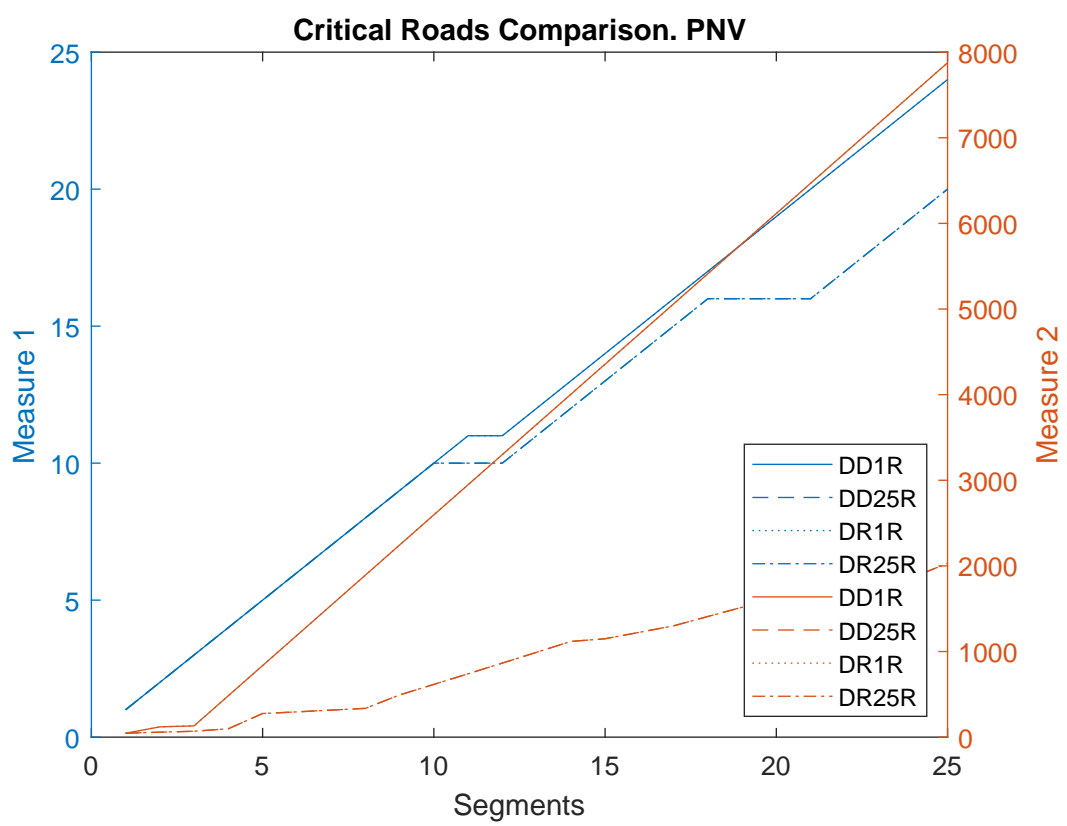

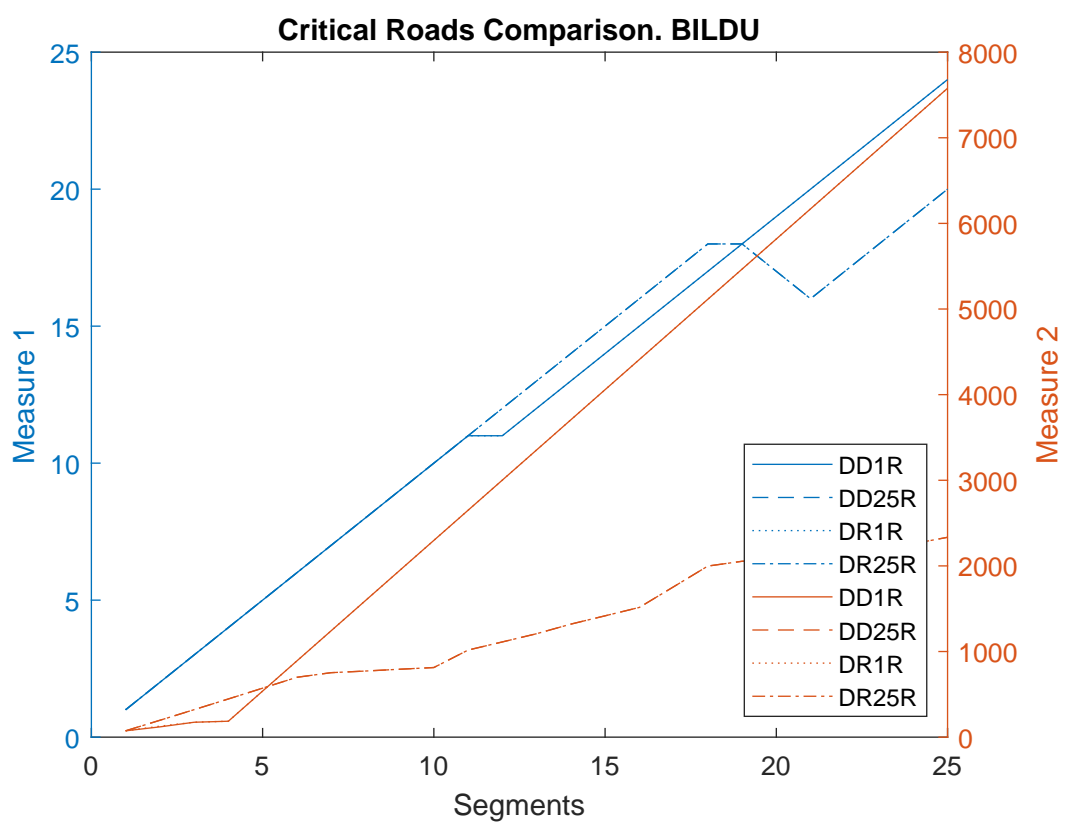

Supplement: Supplementary file 1 — Supplementary file1 (PDF 34 kb) Figures similar to Figure 3 are available online for each of the constituency scenario. See the Appendix. [file 12061_2022_9451_MOESM1_ESM.pdf]
